# Place du traitement chirurgical sous circulation extracorporelle à cœur battant dans les cancers du rein avec envahissement cave supra-diaphragmatique: à propos de sept cas

**DOI:** 10.11604/pamj.2014.19.381.4657

**Published:** 2014-12-16

**Authors:** Mounir Lahyani, Tarik Karmouni, Khalid Elkhader, Abdellatif Koutani, Ahmed Ibn Attya Andaloussi

**Affiliations:** 1 Service d'Urologie B, CHU Ibn Sina, Rabat, Maroc

**Keywords:** Thrombectomie atrio-cave, cancer du rein, circulation extracorporelle, atriocaval thrombectomy, kidney cancer, extracorporeal circulation

## Abstract

Ce travail vise à analyser les résultats de la néphrectomie avec thrombectomie atrio-cave sous circulation extracorporelle (CEC) chez sept patients ayant un cancer du rein avec envahissement cave supra-diaphragmatique et de discuter les indications opératoires. Sept patients, six hommes et une femme dont l’âge varie entre 46ans et 65ans, ont été opérés d'un cancer du rein avec extension atrio-cave. L’écho-doppler a toujours permis la mise en évidence de l'extension veineuse mais la limite supérieure du thrombus était formellement identifiée par l'examen tomodensitométrique quatre fois, et par la résonance magnétique nucléaire dans tous les cas. Tous les patients ont été opérés sous CEC à cœur battant en normothermie. Un seul décès postopératoire est survenu. La durée du séjour en réanimation a été de 4,5 jours. Cinq patients ont eu à distance une dissémination métastatique. Cinq malades ont eu une médiane de survie de 11,5 mois (de 7 à16). Un malade a subi une métastasectomie pulmonaire 6 mois après la néphrectomie. L'exérèse des thrombi atrio-caves a été facilitée par la CEC avec une mortalité et une morbidité postopératoires acceptables mais les résultats à distance ont été décevants. Cette intervention ne peut être proposée qu'aux patients n'ayant aucune extension locorégionale et générale décelable, ce qui souligne l'importance des examens morphologiques préopératoires.

## Introduction

5% des cancers du rein ont une extension tumorale veineuse cave inférieure [1–3], plus particulièrement lorsqu'il s'agit d'un adénocarcinome à cellules claires du rein droit [1, 4, 5]. Ce thrombus néoplasique atteint l'oreillette droite dans moins de 2% des cas [6]. Le traitement carcinologique nécessite l'exérèse du thrombus néoplasique atrio-cave en utilisant une circulation extracorporelle. Les améliorations techniques permettent actuellement de réaliser cette intervention avec une mortalité hospitalière comprise entre 0 et 7% [7–10]. Cependant, la légitimité de ce geste reste controversée et doit être discutée en fonction des résultats à distance. La survie à cinq ans, variant de 17 à 54%, dépend principalement de l'histologie de la tumeur, de son extension, et des caractéristiques de l'extension veineuse néoplasique [2, 5, 10, 11]. Le but de ce travail était d'analyser les résultats chez sept patients opérés d'un cancer du rein avec thrombus néoplasique atrio-cave et de souligner l'importance des examens morphologiques préopératoires à la recherche de certains facteurs pronostiques qui conditionnent l'indication opératoire.

## Méthodes

**Figure 1:**
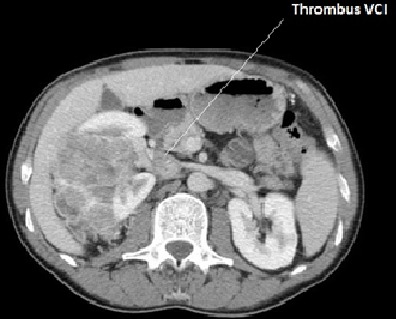
Coupe uro-TDM objectivant la masse rénale droite et le thrombus de la VCI

**Figure 2:**
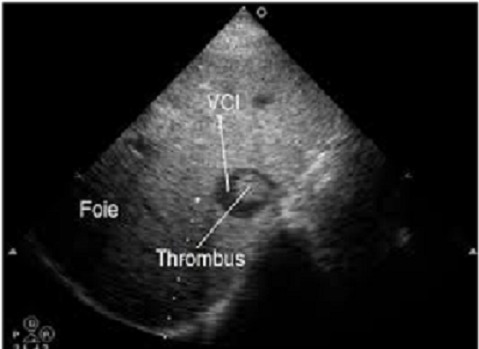
Mise en évidence d'un thrombus cave à l’échographie

**Figure 3:**
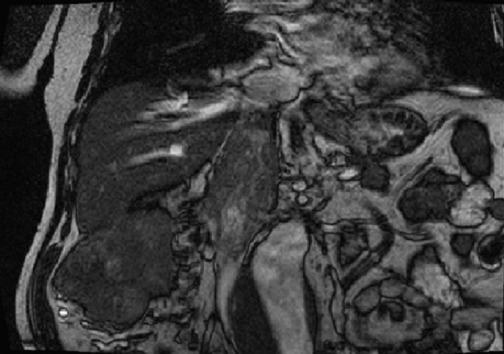
Coupe coronnale IRM précisant lanature néoplasique du thrombus et son étendue

D'octobre 2005 à janvier 2010, sept patients adultes, six hommes et une femme, âgés de 55± 9 ans ont eu une néphrectomie élargie avec thrombectomie cavo-atriale sous circulation extracorporelle pour traiter une tumeur rénale droite: (n = 5), ou gauche: (n = 2) avec thrombus néoplasique. Dans tous les cas, l'extension cave supra-diaphragmatique correspondait à un stade T3c de la classification TNM 97. Pour les sept patients, la symptomatologie évoquait une tumeur rénale: douleur lombaire (n = 5), hématurie (n = 4), altération de l’état général (n = 3), fièvre (n = 2) mais aucun signe spécifique de thrombose cave n’était constaté. L'exploration abdominopelvienne a été faite par l’échographie (n = 7), par la tomodensitométrie (TDM) (n = 5) et par la résonance magnétique nucléaire (IRM) (n = 6). Dans tous les cas, l’échographie abdominale découvrait l'extension cave de la tumeur rénale. La limite supérieure du thrombus néoplasique était formellement identifiée par la TDM dans quatre cas (Figure 1), et par l’écho-doppler et l'IRM dans tous cas (Figure 2, Figure 3). Chez cinq patients, une extension locorégionale était suspectée: adénopathies (n = 3), graisse péri-rénale: (n = 5). L'extension à distance était appréciée dans tous les cas par la radiographie pulmonaire, la scintigraphie osseuse, l'examen tomodensitométrique cérébral et thoracique. Deux patients avaient des nodules pulmonaires à l'examen radiographique (n = 1) et scannographique (n = 1).

TECHNIQUE: Tous les patients ont été opérés avec deux équipes chirurgicales (urologique et cardiovasculaire) par une double voie: une sternotomie a été associée à une laparotomie bi-sous-costale. L'intervention débutait par la dissection abdominale: libération et luxation du foie, décollement de l'angle colique droit et décollement duodéno-pancréatique. Ainsi exposées, la veine cave inférieure sous-rénale, rétrohépatique et les deux veines rénales pouvaient être contrôlées et clampées à la demande. La ligature de l'artère rénale précédait le geste vasculaire. La CEC était installée entre une canule cave supérieure et une canule cave inférieure (n = 5) en amont des rénales ou une canule veineuse fémorale (n = 2). L'intervention était réalisée toujours en normothermie.

**Figure 4:**
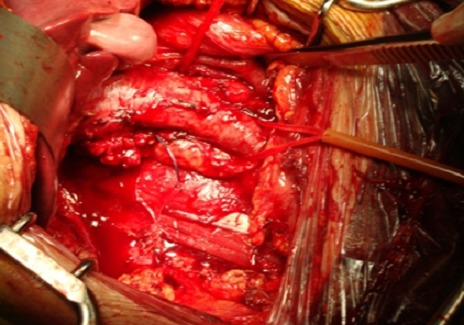
Image per-opératoire montrant l'exposition de la VCI après la néphrectomie totale élargie et juste avant de la cavotomie

La thrombectomie était effectuée par une atriotomie droite associée à la cavotomie, à coeur battant, après clampage des veines caves, supérieure et inférieure sous-rénale, et du pédicule hépatique. La durée moyenne de CEC était de 72 minutes (40 à 210) (Figure 4). Deux fois l'exérèse du thrombus néoplasique imposait une cavectomie partielle en raison d'adhérences à la paroi veineuse. Ensuite, la néphrectomie élargie, emportant la surrénale et les ganglions pédiculaires, était réalisée après neutralisation de l'héparine. Une transfusion a été nécessaire chez tous les patients (3,8 culots/patient).

## Résultats

Un seul patient est décédé en période périopératoire. Trois patients ont eu une évolution postopératoire compliquée: insuffisance respiratoire (n = 1), insuffisance rénale (n = 1), sepsis grave (n = 1). La durée du séjour en réanimation (Tableau 1) a été de deux à cinq jours (moyenne 3,8 jours). L'examen anatomopathologique a montré six adénocarcinomes à cellules claires de type Grawitz et un carcinome de Bellini. Un envahissement locorégional (Tableau 2) était constaté chez cinq patients (graisse périrénale (n = 5), chaînes ganglionnaires (n = 3). La tumeur pesait en moyenne 750 grammes (400 à 1290 grammes).

**Tableau 1:** Technique opératoire et durée de séjour en réanimation

	1	2	3	4	5	6	7
CEC	+	+	+	+	+	+	+
Température CEC	Normothermie	Normothermie	Normothermie	Normothermie	Normothermie	Normothermie	Normothermie
Transfusion	+	+	+	+	+	+	+
Séjour en réanimation (jours)	3	5	4	2	5	4	3

**Tableau 2:** Facteurs pronostiques et survie à distance

	1	2	3	4	5	6	7
Histologie	Grawitz	Grawitz	Grawitz	Grawitz	Bellini	Grawitz	Grawitz
Graisse périrénale	+	+	+	-	+	-	+
Ganglions	-	+	-	-	+	+	-
Métastases pré-opératoires		pulmonaires			pulmonaires		
Récidive locale		Loge rénale					
Métastases post-opératoires	poumon	cerveau	poumon	os	cerveau		
Survie (mois)	11	8	10	16	7	Décès post-opératoire	Toujours vivant

L’évolution (Tableau 2) était marquée par la dissémination de la maladie: métastases pulmonaire(n = 2), cérébrale (n = 2), osseuse (n = 1). Ces localisations secondaires étaient responsables du décès dans un délai inférieur à 11 mois pour quatre patients, au 16e mois postopératoire pour le cinquième. Le dernier malade est tjours vivant. La survie à deux ans des cinq patients qui avaient un envahissement de la graisse périrénale était de 0%. La survie des six patients est rapportée dans le Tableau 2.

## Discussion

Dans ces formes particulières de cancer du rein avec extension veineuse supra-diaphragmatique, la circulation extracorporelle (CEC) rend la néphrectomie et la thrombectomie réalisables avec une mortalité et une morbidité hospitalières acceptables. Nos résultats sont comparables à ceux de la littérature utilisant la même stratégie. La mortalité hospitalière rapportée varie de 0 à 6% [12–14] et la morbidité de 30 à 40% [10, 12]. La CEC permet d'ouvrir l'oreillette droite et toute ou partie de la veine cave inférieure. Elle réduit de façon très importante le risque d'embolie tumorale et permet de contrôler de visu la qualité de la thrombectomie. Elle ne majorerait pas les risques de dissémination cancéreuse, le filtre artériel inclus dans le circuit de CEC semblant capable de retenir les amas de cellules néoplasiques [14]; il n'en reste pas moins que la CEC modifie la réponse immunitaire de l'organisme. L'hypothermie profonde, associée à une période d'arrêt circulatoire a la faveur de nombreuses équipes [6, 7, 14, 15]. Si elle permet de limiter la dissection hépatique et de réaliser la thrombectomie cave dans un champ opératoire complètement exsangue, elle accroît le risque d'insuffisance rénale ou hépatique, de complications septiques et d'accidents vasculaires cérébraux [15]. Le recours à l'arrêt circulatoire en hypothermie profonde n'est pas obligatoire, les aspirations de CEC autorisent la même exposition veineuse, sans augmenter la déperdition sanguine; comme d'autres, c'est l'option que nous avons choisie [12]. L'autotransfusion peropératoire de sang épanché ne semble pas augmenter les risques de dissémination.

Des survies de 17 à 51 mois ont été rapportées chez des patients qui en ont bénéficié [12]. Une autre modalité technique, utilisée par certains, est de réaliser uniquement un circuit veino-veineux entre une veine fémorale et l'oreillette droite, avec la possibilité de transformer ce circuit en CEC complète en cas de nécessité [4]. L'intérêt du circuit veino-veineux sans CEC est d’éviter les conséquences de l'immunodépression liée à la CEC chez ce type de patient. Mais la CEC peut s'avérer indispensable, et de façon imprévisible avant l'exploration chirurgicale de la jonction cavo-atriale, si le thrombus néoplasique est adhérent et s'il faut réséquer une partie des tissus de cette jonction. Enfin, certains auteurs ont proposé de réaliser une embolisation préopératoire de l'artère rénale. Ce geste est controversé; son principal intérêt est de faciliter l'abord du pédicule, d'obtenir un meilleur plan de clivage et ainsi de diminuer les pertes sanguines [16]. Néanmoins, une intervention aussi importante ne se justifie, en dépit d'une mortalité et d'une morbidité opératoires faibles, que si elle améliore le pronostic à long terme de ces patients. Dans le cadre de l'adénocarcinome du rein, trois critères classiques permettent d'appréhender la survie des patients opérés. Ce sont les métastases préopératoires, l'extension à la graisse péri-rénale ou aux chaînes ganglionnaires. Les métastases préopératoires (M +) sont le facteur pronostique le plus péjoratif. La survie des patients M + est de 0% à un an avec une médiane de survie de quatre mois [5, 8]. L'extension locorégionale est également de mauvais pronostic. Pour Hatcher et al. [2], en cas d'envahissement ganglionnaire, la survie actuarielle à cinq ans est de 17% avec une médiane de 0,8 an; en cas d'envahissement de la graisse péri-rénale, la survie à cinq ans est de 11% avec une médiane de 0,9 an. L'extension veineuse, quelle que soit sa limite, n'est pas en elle-même un facteur de mauvais pronostic [1, 2, 17]. Une survie actuarielle de 50% à cinq ans peut être espérée dans le cas où le bilan d'extension locorégionale et métastatique est négatif [3, 18, 19]. De plus, l'envahissement de la paroi veineuse, à condition que la résection soit complète, n'influence pas le pronostic à long terme [2, 20, 21]. Pour certains, cet envahissement de la paroi de la veine cave semble pouvoir être corrélé à un diamètre du thrombus de plus de 40 mm et ainsi être suspecté en préopératoire par les examens morphologiques [22]. Néanmoins, certains auteurs constatent une dissémination métastatique [7, 8] ou un envahissement locorégional [10, 11], chez une proportion importante de patients, lorsque le thrombus cave est rétro hépatique ou supra-diaphragmatique. La néphrectomie et la thrombectomie ne prolongent la survie de façon significative que dans le cas des tumeurs T3c en l'absence d'extension locorégionale ou générale, c'est donc souligner l'importance des examens préopératoires.

L'imagerie préopératoire a un double rôle. Elle permet de détecter l'extension tumorale, de préciser le niveau supérieur du thrombus et son éventuelle adhérence à la paroi veineuse. Tous ces éléments conditionnent la stratégie opératoire. Mais elle doit aussi préciser l’état de la graisse péri-rénale et des chaînes ganglionnaires et éliminer une dissémination métastatique. L’échographie abdominale est, à notre avis, un examen capital: elle est non invasive et peut être répétée à souhait, elle fait le diagnostic de thrombus néoplasique et permet le plus souvent d'en préciser les limites. Bien qu'invasive, l’échographie trans-oesophagienne est très performante dans l'exploration de la veine cave rétro-hépatique et de l'oreillette droite. Elle délimite précisément l'extension du thrombus. Utilisée en per-opératoire, elle permet d’évaluer la fonction ventriculaire gauche et de contrôler la résection du thrombus cave [23]. Les examens, tomodensitométrique et par résonance magnétique nucléaire permettent une analyse morphologique complète: reins, graisse péri-rénale, veine cave inférieure, chaînes ganglionnaires, cerveau et parenchyme pulmonaire. L'intérêt de la TDM par rapport à l'IRM est l’étude fonctionnelle du parenchyme rénal controlatéral. En ce qui concerne l'appréciation de la limite supérieure de l'extension cave, l'IRM a été la plus performante dans notre série. La plupart des auteurs la considèrent comme l'examen de choix tant dans le diagnostic de l'extension veineuse cave que dans l'appréciation de la limite supérieure [4, 8, 9, 24]. La scintigraphie osseuse doit être également réalisée. L’évaluation préopératoire de ces facteurs pronostiques permet de stratifier les indications opératoires: - l'indication est licite dans les cancers du rein non métastatiques, quel que soit le niveau d'extension dans la veine cave, dans la mesure où il est possible de réaliser une exérèse carcinologique sous CEC avec une morbi-mortalité opératoire faible. - la réalisation de la néphrectomie est plus discutable en cas de métastase car il n'est pas démontré que l'opération prolonge la survie des patients. Dans cette situation, seule une symptomatologie invalidante (douleur ou hémorragie) pourrait la faire envisager.

## Conclusion

L'exérèse des cancers du rein avec envahissement cave supra-diaphragmatique est réalisable sous circulation extracorporelle avec un taux de morbidité et de mortalité faible. Les résultats ont été décevants car chez les quatre malades qui avaient tous un envahissement de la graisse périnéale la médiane de survie a été de 9 mois. Cette intervention doit être seulement proposée aux patients n'ayant aucune extension locorégionale ou générale décelable, ce qui souligne l'importance des examens morphologiques préopératoires.
